# JMS: An Open Source Workflow Management System and Web-Based Cluster Front-End for High Performance Computing

**DOI:** 10.1371/journal.pone.0134273

**Published:** 2015-08-17

**Authors:** David K. Brown, David L. Penkler, Thommas M. Musyoka, Özlem Tastan Bishop

**Affiliations:** Research Unit in Bioinformatics (RUBi), Department of Biochemistry and Microbiology, Rhodes University, Grahamstown, South Africa; Swiss Institute of Bioinformatics, SWITZERLAND

## Abstract

Complex computational pipelines are becoming a staple of modern scientific research. Often these pipelines are resource intensive and require days of computing time. In such cases, it makes sense to run them over high performance computing (HPC) clusters where they can take advantage of the aggregated resources of many powerful computers. In addition to this, researchers often want to integrate their workflows into their own web servers. In these cases, software is needed to manage the submission of jobs from the web interface to the cluster and then return the results once the job has finished executing. We have developed the Job Management System (JMS), a workflow management system and web interface for high performance computing (HPC). JMS provides users with a user-friendly web interface for creating complex workflows with multiple stages. It integrates this workflow functionality with the resource manager, a tool that is used to control and manage batch jobs on HPC clusters. As such, JMS combines workflow management functionality with cluster administration functionality. In addition, JMS provides developer tools including a code editor and the ability to version tools and scripts. JMS can be used by researchers from any field to build and run complex computational pipelines and provides functionality to include these pipelines in external interfaces. JMS is currently being used to house a number of bioinformatics pipelines at the Research Unit in Bioinformatics (RUBi) at Rhodes University. JMS is an open-source project and is freely available at https://github.com/RUBi-ZA/JMS.

## Introduction

Computational pipelines or workflows have become an important tool for the analysis of the vast amounts of data being generated in many scientific fields today. The computational complexity of these workflows varies significantly, but can often require days of computing time and a large amount of computing power. To speed up the execution of these jobs, the use of parallel algorithms and high performance computing (HPC) clusters has become increasingly common. Computer clusters offer high performance through the aggregation of resources from multiple individual computers. Resource managers are software systems that are required to manage the submission and scheduling of jobs on these clusters as well as the allocation of resources, such as memory and processing cores, to individual jobs. Examples of this type of software include the Torque resource manager [1] as well as the Simple Linux Utility for Resource Management (SLURM) [2]. These systems provide fine-grained control over the resources of a cluster, allowing users to configure and manage nodes, submit jobs, and administer their systems. They can also be integrated with 3^rd^ party job schedulers such as Maui [3] for improved job scheduling capabilities. Unfortunately, in most cases, the powerful advantages of running parallel applications on HPC clusters remain out of reach of the average user. As computational modelling and big data analysis gain popularity in a wide range of fields, more and more researchers are requiring the use of HPC resources. These researchers often have little or no command-line expertise. Resource managers such as Torque and SLURM are command-based and present a steep learning curve to such users, often preventing them from making use of the computing resources at all.

To make HPC computing more accessible to all scientists, fully-functional, click-based interfaces are required. One such system is CHReME [4]. CHReME provides a web-based interface to Torque and, amongst other things, facilitates the submission, management and monitoring of jobs on the cluster as well as user and cluster management and configuration. Unfortunately, CHReME is a proprietary system and is not freely available. There are many examples of other similar systems, the Moab suite of HPC applications [5] being particularly noteworthy, but the costs involved in obtaining these systems put them out of reach of many labs. Although some free web interfaces have been developed, these systems usually only provide accounting information for the cluster or allow users to monitor the status of already running jobs. They do not allow users to submit jobs or access results. An exception to this is Yabi [6]. Yabi provides an online environment for creating workflows and manages the execution of those workflows in an HPC environment. It supports a number of resource managers and records a detailed job history for all jobs run through the interface. Yabi is capable of executing any tool that is available from the command line. Administrators are able to add tools to the Yabi web interface by entering in data describing how those tools can be run from the command line.

Resource managers are only part of the challenge, however. Jobs that are submitted to a cluster are often part of a sequence of jobs that are being run to perform some kind of analysis. When the output of one job becomes the input of another job, this is known as a computational pipeline or workflow. It has become increasingly popular to use software to automate the execution of these workflows instead of micro-managing each job in the sequence. Yabi is one such system capable of doing this. In the biological sciences, a number of additional workflow management systems (WMS) have been developed. Two such systems, Galaxy [7] and Ergatis [8], allow users to create workflows by piecing together a number of tools that come pre-packaged with the systems. Users are also able to add additional tools by creating configuration files describing how these tools can be run. Unfortunately, these configuration files can be difficult for new users to master.

While WMSs like Ergatis and Galaxy manage the execution of tools and scripts directly on the servers or clusters that they are set up on, there is another class of WMSs that creates pipelines out of web services. One example of this type of WMS is Taverna [9]. Taverna takes advantage of thousands of bioinformatics web services that have been developed over the past few years [10] by combining them, along with local scripts, into complex pipelines. The advantage of such an approach is that most of the computation is performed on the remote servers where the web services are hosted, thus, reducing local infrastructure and maintenance costs. Limitations of this approach include bandwidth constraints, as data must be transferred to and from the remote servers, as well as varying reliability of the remote web services.

Systems like Galaxy and Ergatis are capable of running on a cluster, but they provide no visible means of directly interacting with the underlying resource manager. In addition to this, researchers who develop workflows often want to make these workflows available via their own web servers. A workflow created in Galaxy, for example, can only be run from the Galaxy web interface. In order to integrate computational pipelines into their own web servers, developers must spend time building a user-friendly interface and managing the flow of data from the interface to the server and then finally onto the cluster.

In this paper, we introduce the Job Management System (JMS). JMS combines the functionality of a web-based cluster front-end with that of a WMS. It exposes this functionality via a RESTful web API [11], allowing developers to include an interface to workflows that have been created within JMS in their own web servers or simply access them from within their own scripts. JMS also provides the ability to add new tools and scripts directly via the web interface, without any need for complicated configuration files. JMS aims to eliminate the development barriers involved in making tools and scripts available via public web interfaces by allowing developers to create and run these tools over a cluster without requiring the developer to have any HPC background. Once tools have been created, a user-friendly, web-based interface is automatically generated to facilitate the use and execution of the tools by non-IT experts.

Although the functionality of JMS is applicable to any scientific field, it is currently being tailored towards bioinformatics with the introduction of bioinformatics related tools, workflows and result editors. The system is being developed for use by the H3Africa Consortium [12]. As part of this Consortium, numerous groups around Africa have received funding to purchase high-end servers for use in computationally intensive bioinformatics analyses. JMS can be used to assist these groups with the initial setup stage as well as managing and monitoring their servers and developing and managing tools and workflows. Reporting tools will allow groups to monitor their cluster usage and report back to funders and other stakeholders on their activities. JMS will further provide a work environment to share tools, scripts and workflows between collaborating groups. In these ways, JMS can be expected to enhance progress towards the Consortium’s scientific goals.

## System Design & Features

### Implementation Details

JMS was developed using the Django web framework [13] on the Linux distribution, Ubuntu 12.04. The web server chosen to host the system was Apache2. JMS requires a relational database to store user, workflow and job information. During development, a SQLite database was used, but in production we recommend a more heavyweight option such as MySQL. Since the Django object relational mapper (ORM) handles the database interactions, any other Django supported database management system (DBMS) can be used. Support for resource managers is added via the development of plugins. Currently plugins for Torque and SLURM exist and a plugin for the Univa Grid Engine (UGE) [14] (previously known as the Sun Grid Engine [15]) is being developed. The Torque resource manager was initially used to handle job submissions and scheduling during development. Previous HPC systems, such as WImpiBLAST [16], have similarly integrated Torque into their architectures. The JMS interface is web-based and was developed using the Knockout.js [17] and Bootstrap [18] frameworks.

### Architecture

There are two ways in which one can describe JMS architecture. The first is by looking at the JMS application itself. This will be referred to as the *software architecture* of JMS. The second is by looking at how JMS slots into the existing HPC cluster architecture. This will be referred to as the *system architecture*.

#### 1.1 Software Architecture

**Fig 1:**
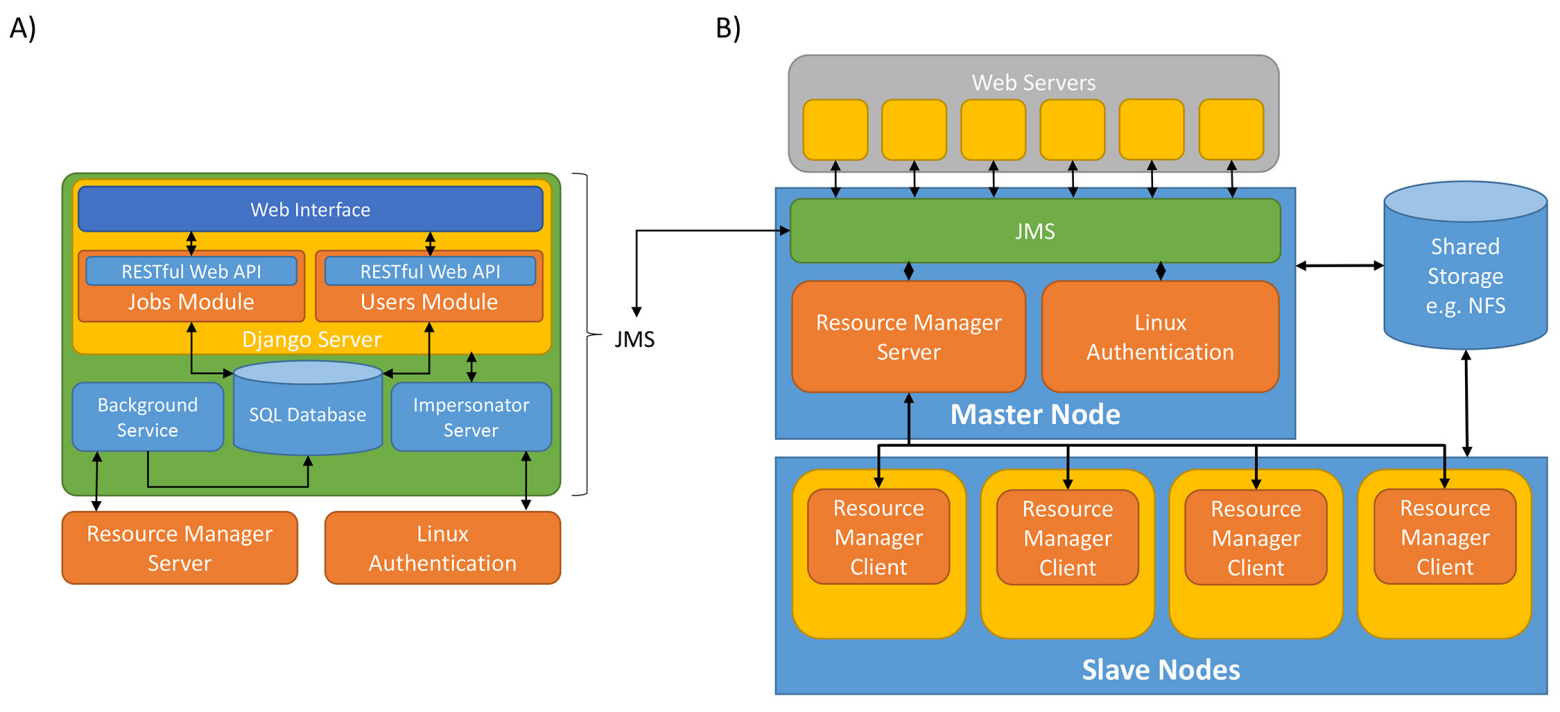
JMS System Architecture. A) JMS has been developed as a Django web application. The project consists of three modules, a background service, an impersonator server and a SQL database. The background service is used to update job history in the database. The impersonator server forms part of the security system and allows the JMS to impersonate users on the cluster. The *jobs* module is the main module and is responsible for interfacing with the resource manager as well as providing the WMS functionality. The *users* module is responsible for handling user authentication and security. It also provides basic social networking functions. Both these modules expose their functionality via a RESTful web API. The interface module makes use of these web APIs to provide a web-based interface for the system. B) JMS forms part of a broader architecture. It provides an interface for external web servers to run jobs on a cluster. Authentication is done via the Linux authentication system so that users have the same permissions they would have if they were to log into the server via SSH.

The JMS web application has been developed using the Django web framework (Fig 1A), and provides a web-based interface and WMS for the Torque resource manager. The application adopts the traditional, three-tiered architecture commonly used for web applications and consists of a presentation layer, application layer, and a data storage layer.

The presentation layer is responsible for receiving user input and passing it on to the application layer. JMS provides a user-friendly web interface that has been built using the Knockout.js and Bootstrap frameworks. The interface is responsive, meaning that it adapts according to the size of the screen. As such, JMS is user-friendly on a wide range of devices and allows users to monitor and manage jobs on their mobile device when out of the office.

Interaction between the web interface and the server is achieved by sending AJAX requests to a RESTful web API. AJAX allows data to be sent to and retrieved from the server without requiring entire pages to be reloaded. Apart from saving bandwidth, this provides a smoother and more fluid user experience. Using a web API to expose the functionality provided by the application layer allows developers to interact with JMS programmatically. This also means that 3^rd^ party developers will be free to create their own, custom interfaces for JMS without needing to modify any JMS code. Similarly, it will allow us to develop additional interfaces for other platforms in the future e.g. mobile apps and desktop clients. The RESTful web API also makes it easy to include workflows that have been set up via JMS in external web interfaces. This negates the need to create entirely new systems each time a researcher wants to provide a web portal to a newly developed tool or workflow.

The application layer consists of two Django modules (or apps), namely, the *users* module and the *jobs* module, and is responsible for performing tasks based on input received from the presentation layer. The *users* module is responsible for user management and authentication and performs a number of security functions, which will be discussed later. It is also responsible for providing the collaboration features built into JMS. The *jobs* module is the biggest module in JMS and is responsible for providing all functionality related to creating and managing workflows, submitting, managing and monitoring jobs and job history, and managing, interacting and configuring resource manager settings via resource manager plugins (discussed later).

In addition to the two Django modules, the application layer also includes a background service and what we have called the Impersonator Server. The purpose of the background service is to continuously poll the underlying resource manager to ensure that job history is kept up-to-date in the JMS database. The Impersonator Server is used to allow JMS to spawn processes on the cluster as another user. This is discussed in more detail in section 5.

The data storage layer is responsible for storing job, workflow, and user details as well as resource manager configuration data and settings. The system was designed and built using the SQLite DBMS, but, because the Django ORM handles interactions with the database, SQLite can easily be substituted by any other relational DBMS supported by the Django framework.

#### 1.2 System Architecture

JMS is installed and configured as a separate component on top of an existing HPC cluster to form a two-tier system architecture (Fig 1B). This means that the existing HPC software (e.g. Torque, SLURM, UGE, etc) can remain as is and does not need to be modified or adjusted in any way. In an effort to make JMS compatible with as many resource managers as possible, we have devised a custom plugin architecture. Using this architecture, adding support for additional resource managers becomes as trivial as writing a Python wrapper for the resource manager. If Python wrappers already exist for the resource manager, this becomes even easier. These wrappers, which we will refer to as plugins, must follow three simple rules.

Firstly, plugins must inherit from our base resource manager class (*BaseResourceManager*.*py*). This class provides a handful of important functions required by JMS, but also requires plugins to override a number of unimplemented functions.

Secondly, a number of predefined objects have been created to serve as the outputs for the base functions that plugins must override. Essentially, plugins must populate these objects with data from the resource manager and return them to JMS. By doing this, JMS receives an object or list of objects that it knows how to deal with. It doesn’t matter that the content of the objects may differ depending on the underlying resource manager, because the objects also contain metadata that describes what type of data they hold. This allows JMS to adapt its interface to best suit the data.

And lastly, plugins must be copied and pasted into a specific folder where JMS knows to look for them. To use a plugin, the plugin name must then simply be specified in the JMS *settings*.*py* file.

Currently, plugins for Torque and SLURM have been developed and development of a plugin for UGE is underway. Using this architecture, we will be able to quickly increase the number of supported resource managers.

### Features

JMS provides features for three types of users, namely, developers, administrators, and researchers. These features fall under a range of categories and are described below.

#### 1.3 Job Management

JMS allows users to submit new jobs to the cluster, monitor and manage jobs while they run, and obtain the results of the jobs once they are complete. It does this by interfacing with the underlying resource manager, as well as the WMS, which will be discussed in the following section.

JMS allows users to upload or create scripts to be submitted to the cluster and then request resources such as required memory, number of cores, and the wall-time to be allocated to the job. Based on these inputs, JMS generates a job script and submits it to the resource manager to be executed. JMS is capable of running any program or script that can be executed from the command-line. Jobs can then be monitored until their completion. What the JMS monitors will be dependent on the resource manager plugin, but will usually include the resources used, the input and output streams of the job, and the working directory of the job. The exit status of the job combined with the output stream can be used to determine whether execution was successful, and, if not, what went wrong. On completion, the user can access the results of a job, either from the output and error streams, or by downloading the resultant files from the working directory. All results can be accessed via the web interface via the Job History tab.

Real-time monitoring of jobs is done by interfacing with the resource manager plugin. Using the Torque plugin as an example, the ‘qstat’ command is used to check for updates on the job. This command is polled continuously by the background service to update job details. Data returned is parsed and stored in the JMS database in order to keep a permanent record of all jobs.

In addition to monitoring the status of jobs, JMS provides users with job management functionality. Users may delete jobs from their job history, cancel running jobs, suspend or hold jobs before or after they start running, and, in future, users will be able to make requests to alter jobs. Because alteration requests may include requests for additional resources, they require an admin user’s approval. If a non-admin user makes a request for an alteration to one of their jobs, the request will be forwarded to an admin user to be granted or denied.

#### 1.4 Workflow Management

In addition to interfacing with the underlying resource manager, JMS provides functionality allowing users to build and execute complex computational pipelines or workflows. Using the JMS model of a workflow, a workflow is made up of a set of stages. Each stage represents a tool that has been added to JMS.

**Fig 2:**
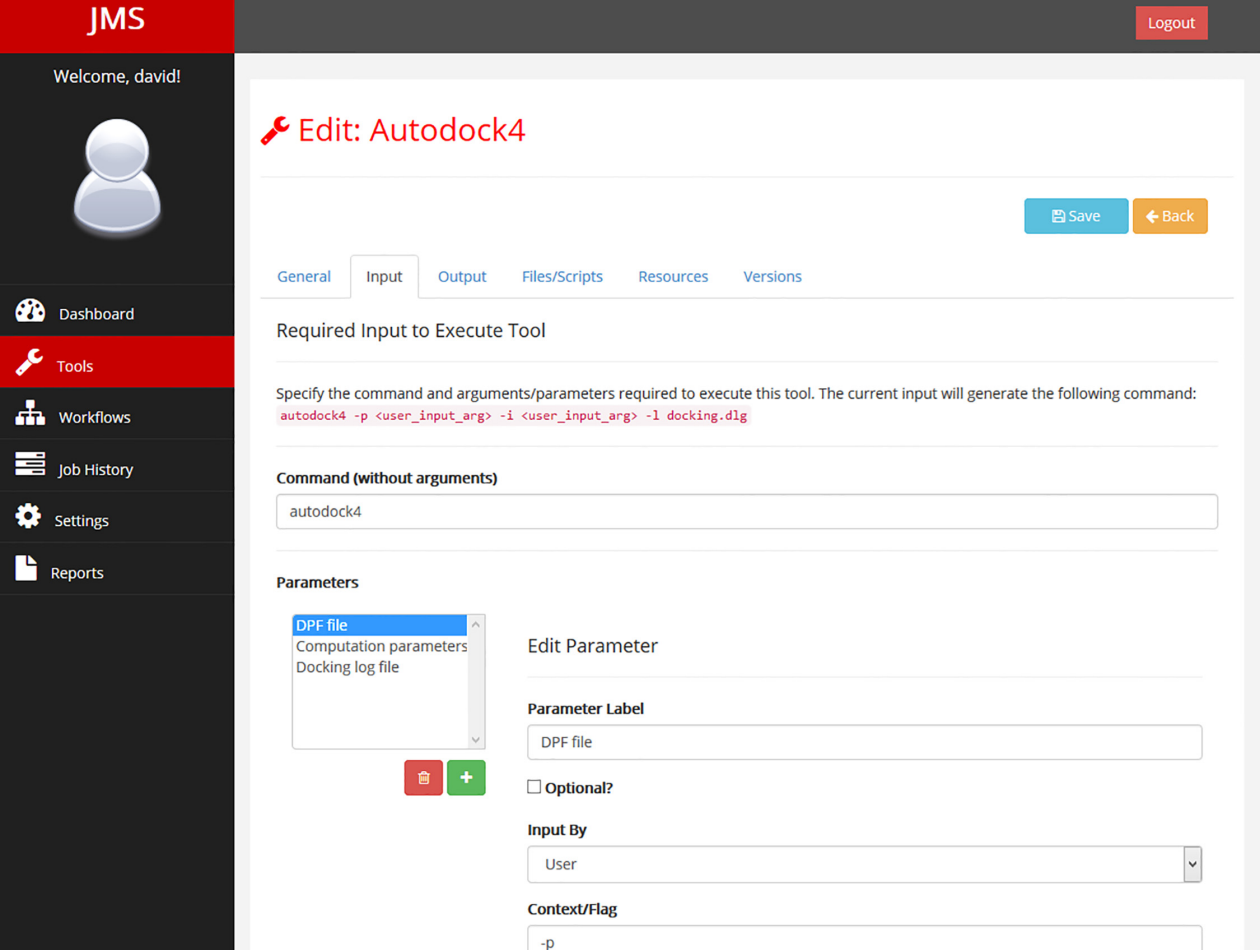
The tool creation interface. JMS provides a user-friendly tool creation interface. Users can name and describe their tools, enter the command and parameters that would be needed to run the tools, specify the expected outputs that the tools would produce, specify the resources that should be allocated to the tool on the cluster, and publish new versions of the tool.

A tool can be any command-line utility that is already installed on the cluster or a custom script or executable that is uploaded by the user. For each tool, users provide JMS with a number of details including the command that would be used to run the tool or script from the terminal, the parameters that the command can take, the resources that should be allocated to the tool by the resource manager, and the expected outputs that the tool will generate. All these details are entered into the system via the web interface (Fig 2) and then stored in the database backend. No complicated configuration files are required. Furthermore, scripts and executables that are uploaded by users are automatically stored in a strict directory hierarchy that is managed by JMS. In addition to uploading scripts, users can create new scripts from scratch via the web interface. Scripts can also be edited via the web interface using a web-based code editor that has been incorporated in JMS. This allows users to update their scripts without needing to re-upload them and introduces a unique ability to troubleshoot scripts and workflows without ever having to leave the web interface. Developers can test their workflows and check the output streams for error messages. They can then edit their scripts based on any error messages returned. Simple bugs, such as syntax errors, often return a message that includes the line number in the script that the error is located on. The developer can then open the script for editing within the browser and fix the mistake before running the workflow again. There is no need to download the script to fix the bug and then upload the updated script again. The code editor is user friendly and provides syntax highlighting for most languages, automatic indentation, and word completion functionality, which often makes it a superior alternative to developing on one’s own machine.

**Fig 3:**
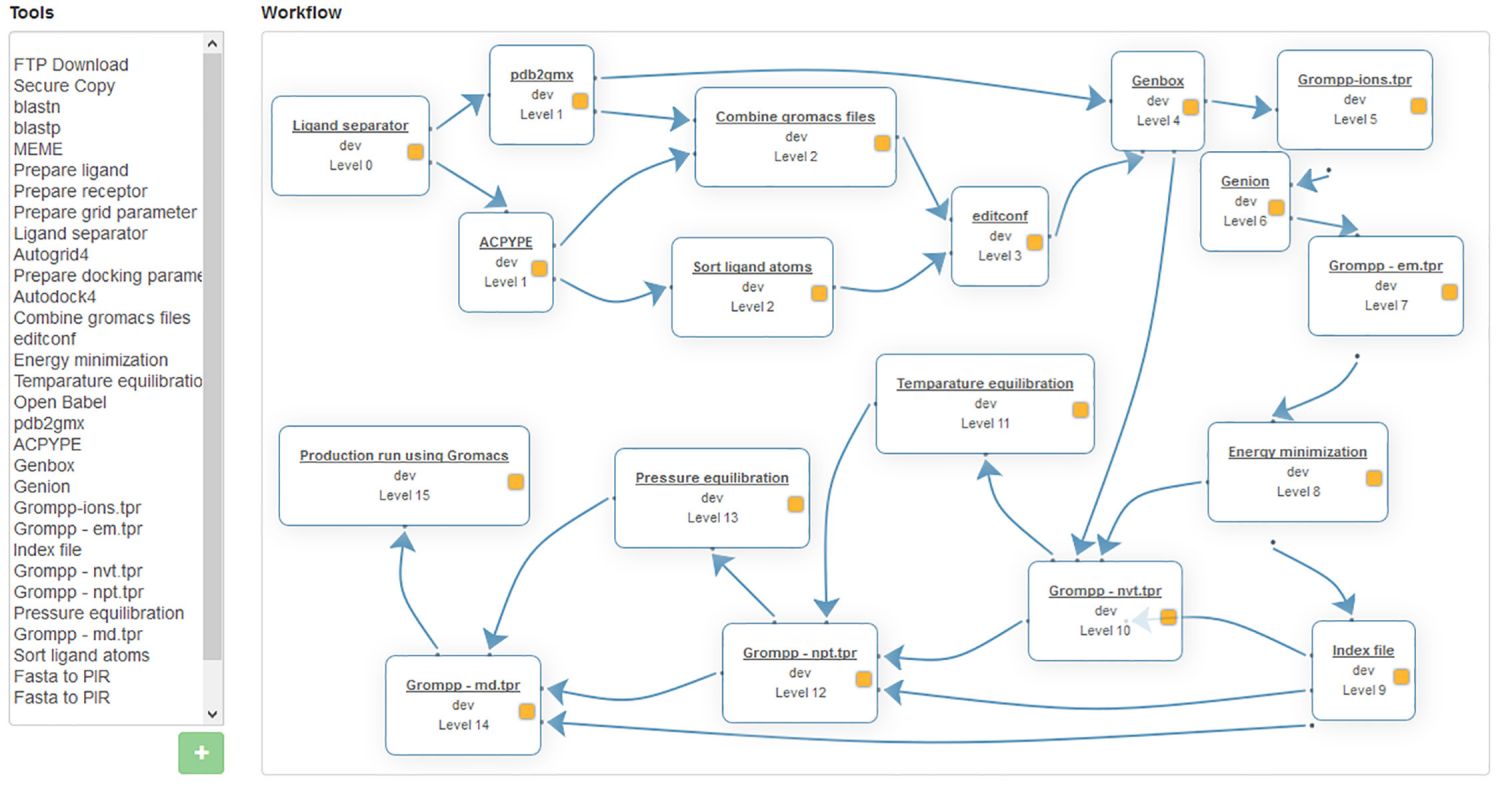
The workflow creation interface. Workflows can be created by double clicking on tools in the list on the left-hand side of the page and arranging them on the canvass provided. Relationships/stage dependencies can be created between tools by dragging a line from one tool to another. Stages can be edited by double clicking on the tool on the canvas.

JMS provides a simple to use workflow creation interface (Fig 3). A canvas is provided, similar to that provided by Galaxy, where tools can be arranged into complex pipelines. Tools can be added to the canvas from a list on the left hand side of the interface (depending on the size of your screen). Once on the canvas, they can be rearranged and dependencies can be created between them by dragging and dropping.

**Fig 4:**
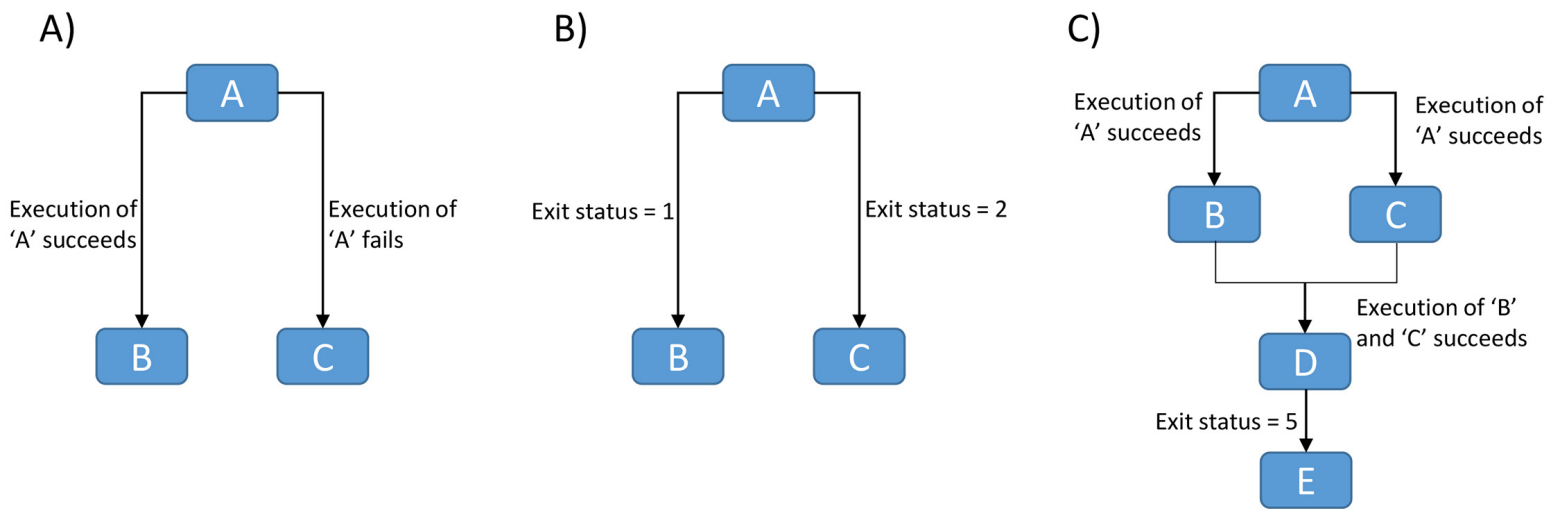
Workflow patterns. A) Stage B is executed if stage A executes successfully, else stage C executes. B) Stage B will only execute if stage A exits with a status code of 1 and stage C will only exit if stage A exits with a status code of 2. If the status code is neither 1 nor 2, the job fails. C) All other stages wait while stage A executes. On successful completion of A, stage B and C execute in parallel. If both stages execute successfully, stage D executes. If stage D exits with a status code of 5, stage E executes.

JMS makes it easy for users to develop complex workflows by creating conditional stages. This is done by setting conditions on stage dependencies. When a dependency is created between two stages by dragging and dropping a line between those stages, a condition can be set for that dependency. For example, given three stages, A, B, and C, a user can set B to run if A completes successfully and C to run if A fails (Fig 4A). The JMS extends this further by allowing users to create dependencies based on the exit status of a stage. For example, B executes if the exit status of the job is 1 and C executes if the exit status of the job is 2 (Fig 4B). Users can take advantage of this functionality by manually setting the exit status of their scripts based on some condition. Using exit statuses to create conditional forks was chosen as it provides limitless possibilities for what developers can do with it. For example, a developer can create a script that has the sole purpose of exiting with a specific status based on user input. If the user is given a list of options, and chooses option 2, the script can accept that input, exit with a status of 2, and JMS will then execute whichever tool was dependant on that stage exiting with a status of 2. Using this logic, there is no workflow that cannot be created within JMS.

JMS also allows for certain stages in a workflow to run in parallel while others are required to run sequentially. For example, if we have stages A to E, we could have a scenario where B and C are dependent on A executing successfully, D is dependent on B and C executing successfully, and E is dependent on D exiting with a status code of 5 (Fig 4C). In such a case, all stages would wait while A executes. On successful completion of A, B and C would both execute and run in parallel. On successful completion of both B and C, D would begin execution. If D exits with a status code of 5, stage E will begin executing. In this example we see that certain stages must execute sequentially, while others (B and C) may execute in parallel.

**Fig 5:**
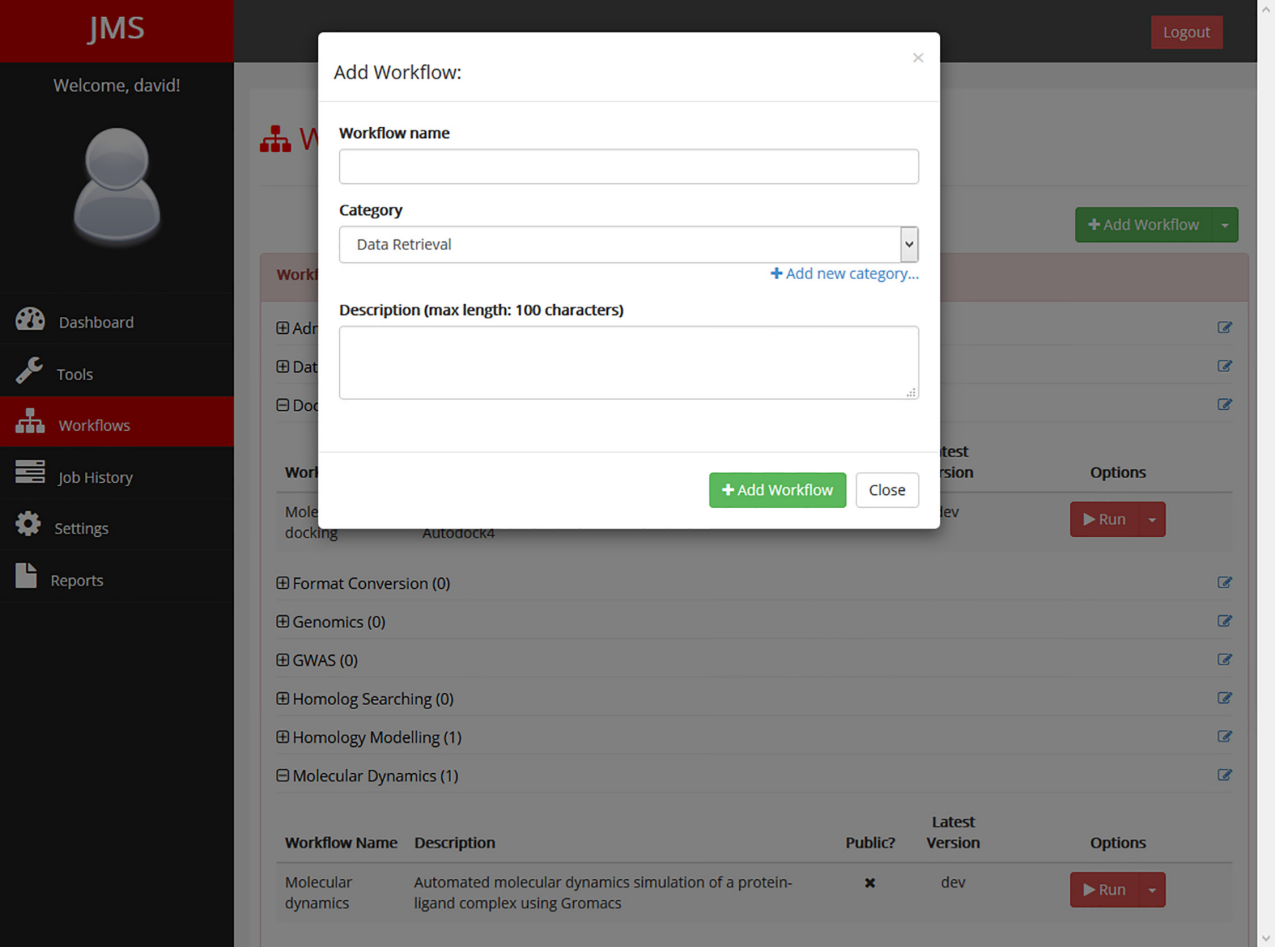
Workflows. The workflow tab displays all workflows that the logged in user has access to. From here, the user can create new workflows and edit, run, or share existing workflows. Workflows can also be imported and exported from this interface.

Workflows and tools can be selected and executed from the respective ‘Workflows’ and ‘Tools’ tabs of the JMS interface (Fig 5). To run a tool or workflow, JMS provides an automatically generated web interface. This interface allows users to enter in values for each of the parameters specified during the creation of the tools. Although other WMS also provide automatically generated interfaces, JMS goes a step further by allowing these interfaces to be integrated into other web servers. A JavaScript plugin is currently under development that will automate the process of generating an interface for a 3rd party website.

Some workflows may consist of a number of different stages, each of which requires a number of different parameter values to be input. To make the process of inputting parameters faster and easier, as well as more consistent, JMS allows users to create input profiles. An input profile can be defined as a set of default inputs for a tool or workflow. Users can create multiple input profiles for each tool or workflow. This is especially useful when a user wants to run a workflow multiple times, where each time only one or two parameters need to be changed. In such a case, an input profile can be created that automatically fills in values for the parameters that are to remain constant so that the user only needs to fill in values for the parameters that will change. Input profiles can also assist novice users by providing them with default inputs for advanced parameters, while expert users will still be free to modify these values.

Although input profiles will significantly speed up the rate at which multiple jobs can be submitted, this may still not be good enough for cases when hundreds or thousands of jobs need to be submitted at once. JMS caters for these cases by allowing users to submit batch jobs. Batch jobs require the user to generate a batch file consisting of rows of parameters, where each row represents a single job. JMS reads in the file and submits each job, one at a time.

The job history stored for tools and workflows is also enriched by JMS. In addition to the cluster-related information obtained from the resource manager, all the data for each stage of the workflow run is stored. This includes all the parameter values that were entered by the user, as well as a snapshot of the working directory after each sequential stage. By storing these details, JMS is able to rerun a workflow from almost any stage.

**Fig 6:**
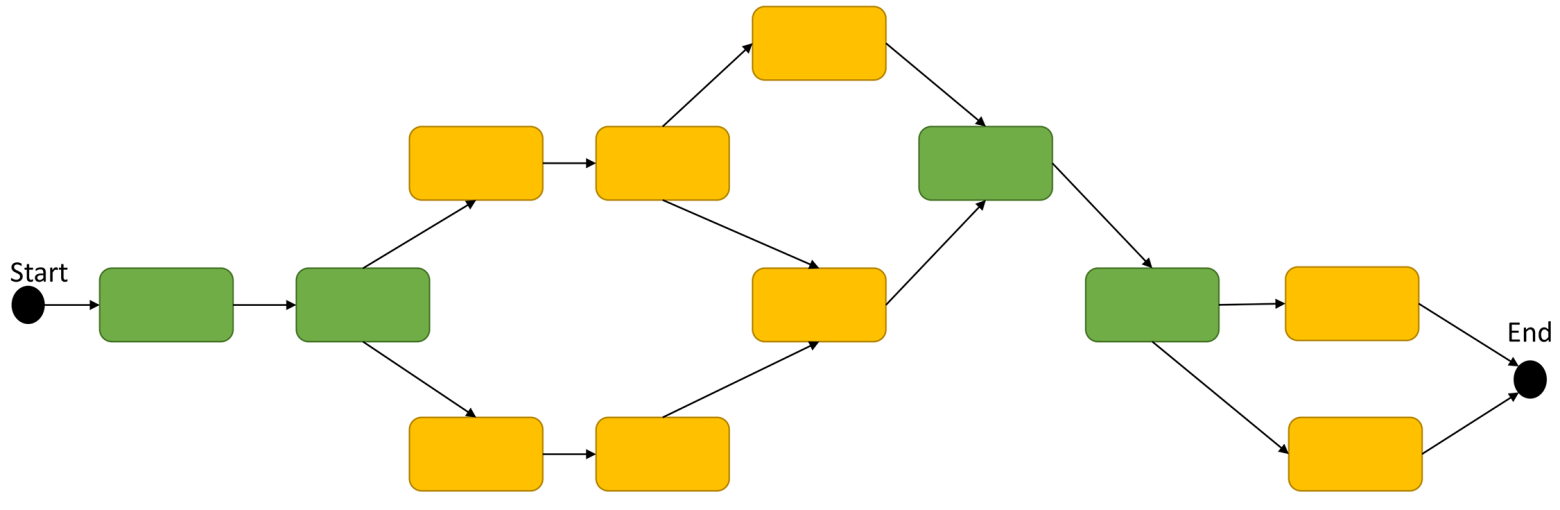
Checkpoints. In the example workflow depicted here, stages that are eligible to act as checkpoints are coloured in green. Only stages that are not running in parallel with any other stages can be used as checkpoints.

Checkpoints are stages within a workflow that a job can be repeated from. Users can specify which stages should be regarded as checkpoints during workflow creation. When JMS reaches that stage during execution of a workflow, it will create a snapshot of the working directory at that point in time. To repeat a job from a checkpoint, JMS simply restores the working directory to the state it was in at that point in time and resubmits jobs using the job history details stored in the database. Not all stages are eligible to be checkpoints, however. Only a stage that does not run in parallel with any other stages can be used as a checkpoint. This is because it is only possible to restart a job from a point where all forks in a workflow have converged (Fig 6). A snapshot of a directory taken at the beginning of a stage will be in the required state to repeat that particular stage, but will not necessarily be in the correct state to repeat a stage that is currently busy executing in parallel.

**Fig 7:**
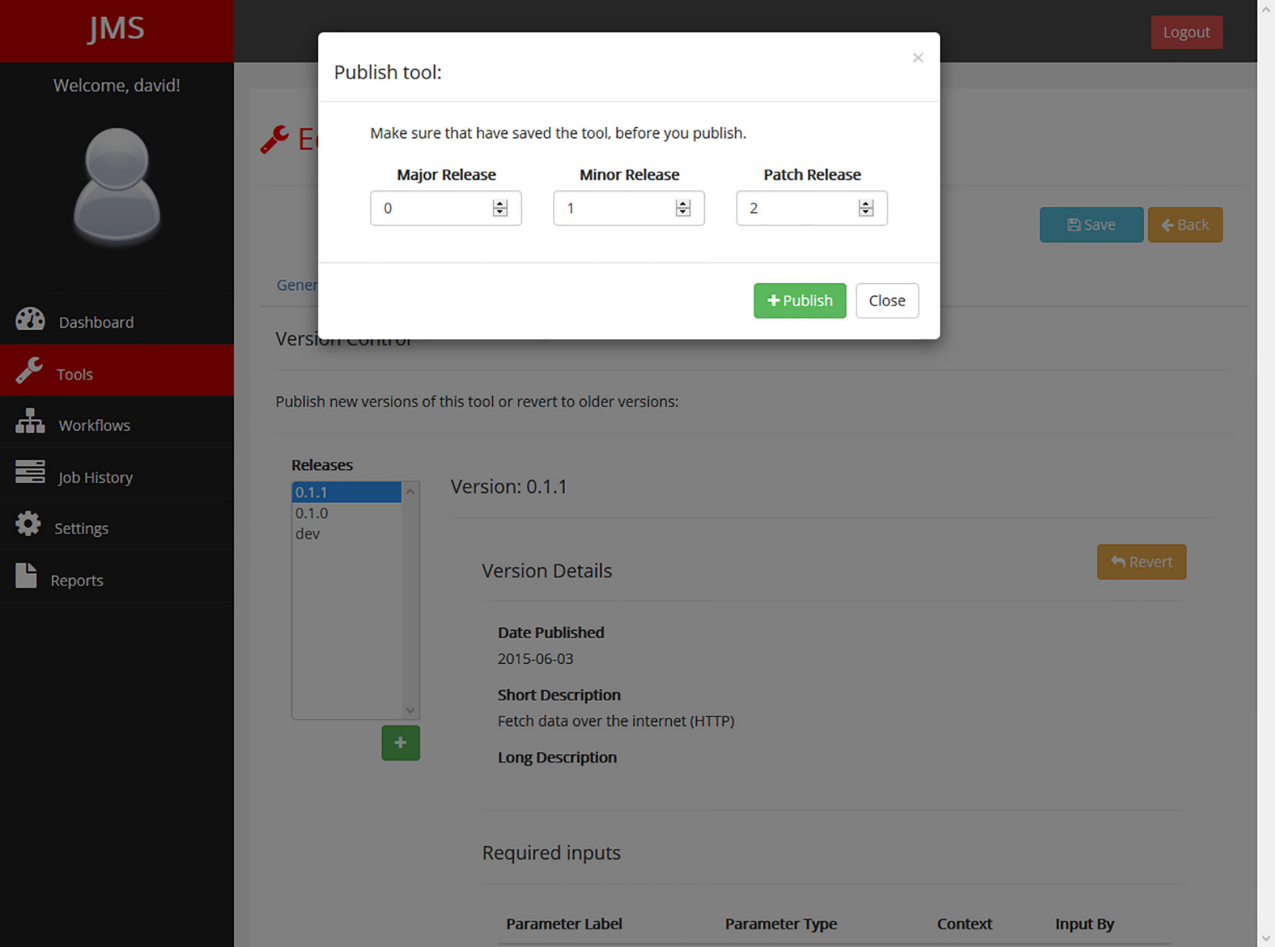
Tool versioning interface. Users may publish a new version of a tool by adding a release or revert the development version to an older version by selecting the version and clicking on the “Revert” button.

A robust versioning system has been incorporated in JMS (Fig 7). A single tool to can have multiple versions. When users first create a tool, a development version is also created. This version is what the user is editing and updating via the web interface. Once the user is happy with the state of their tool, they can publish an official version. This new version is essentially a snapshot of the development version of the tool at that point in time. Everything about the tool including any scripts that have been uploaded are copied and stored separately to the development version. From that point in time, that version of the tool can never again be edited. The user can, however, continue developing and editing the development version or even revert the development version back to one of the previously published versions, and, when ready, publish a new version. Once again, the new version will be a snapshot of the development version at that point in time. As such, the user is always working on the development version of the tool and can never affect any changes to older versions of the tool. This becomes vitally important if a specific version of a tool is used by a workflow.

Workflows use the same versioning system as tools whereby the user is always working on the development version and creates snapshots of the development version at certain points in time to create versions. This allows different versions of a workflow to use different versions of tools. As with tool versioning, once a particular version has been published, it can never again be edited.

JMS encourages developers to use the Semantic Versioning specification [19] for numbering versions. This specification describes a version number using the format MAJOR.MINOR.PATCH, where a major version number change requires an update that makes incompatible changes to the API, a minor version number change is used when an update adds functionality that is backward compatible, and a patch number change is a bug fix.

JMS versioning can be best used in combination with a system such as GNU modules. In such a case, a tool version within JMS could load a GNU module for a specific version of software. The next version of the tool within JMS could be set to load a different GNU module and thus a different software version.

#### 1.5 Dashboard

**Fig 8:**
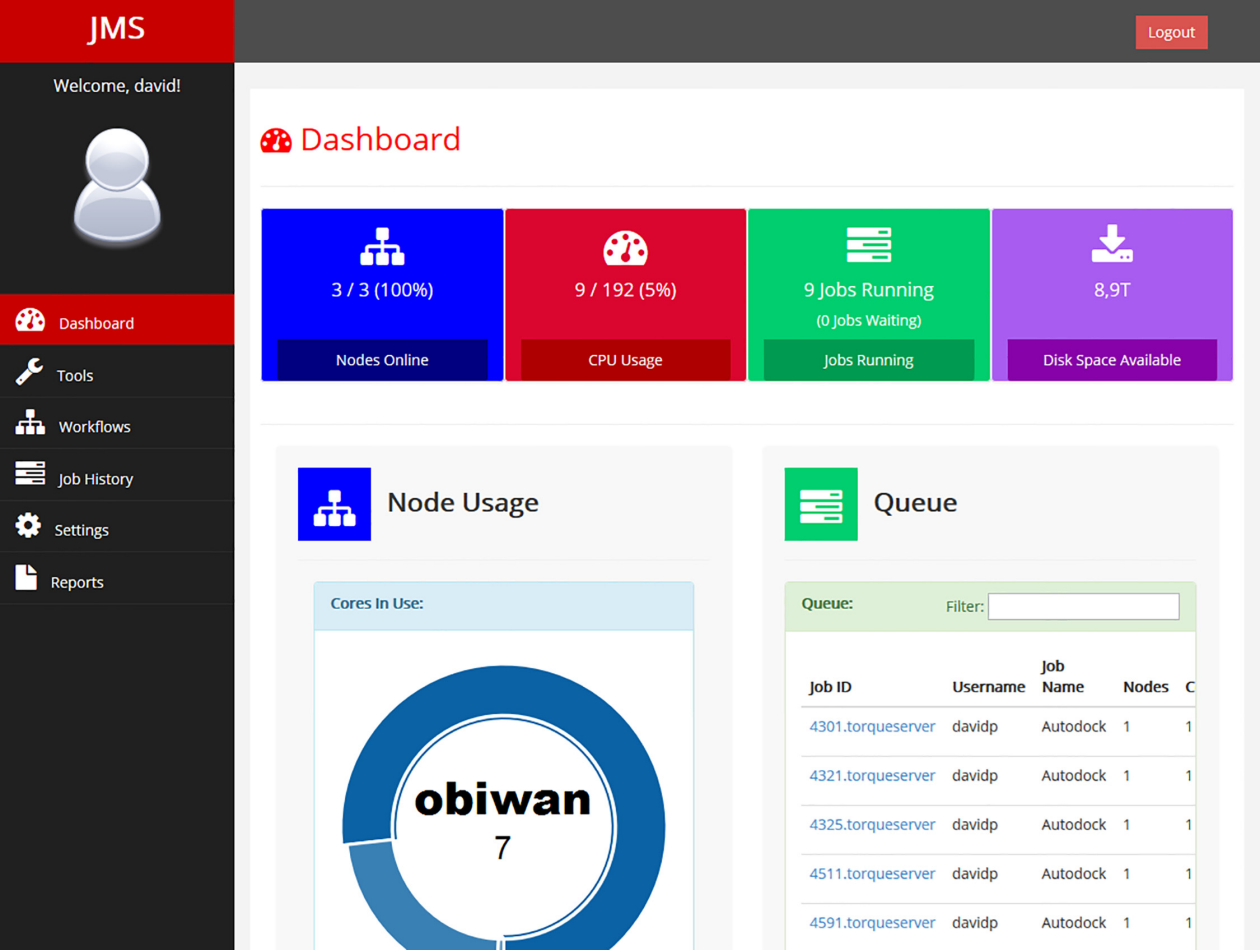
Dashboard. The JMS home page is a dashboard displaying status information for the cluster. The above diagram is a screenshot of the dashboard when the Torque plugin is being used.

JMS provides a dashboard containing detailed status information (Fig 8). Amongst other things, the dashboard provides users with summary information depicting the current state of the cluster. This information includes how many nodes are online/offline, the proportion of processors being used across the whole cluster, the number of jobs currently running or waiting to run, and the amount of disk space still available.

The dashboard also allows users to check the state of each node in the cluster as well as the current queue of jobs submitted to the cluster. The specific information displayed here will depend on the resource manager plugin being utilised. If users have sufficient privileges, they will be able to cancel the job directly from the queue.

#### 1.6 Access Control & Collaboration

**Fig 9:**
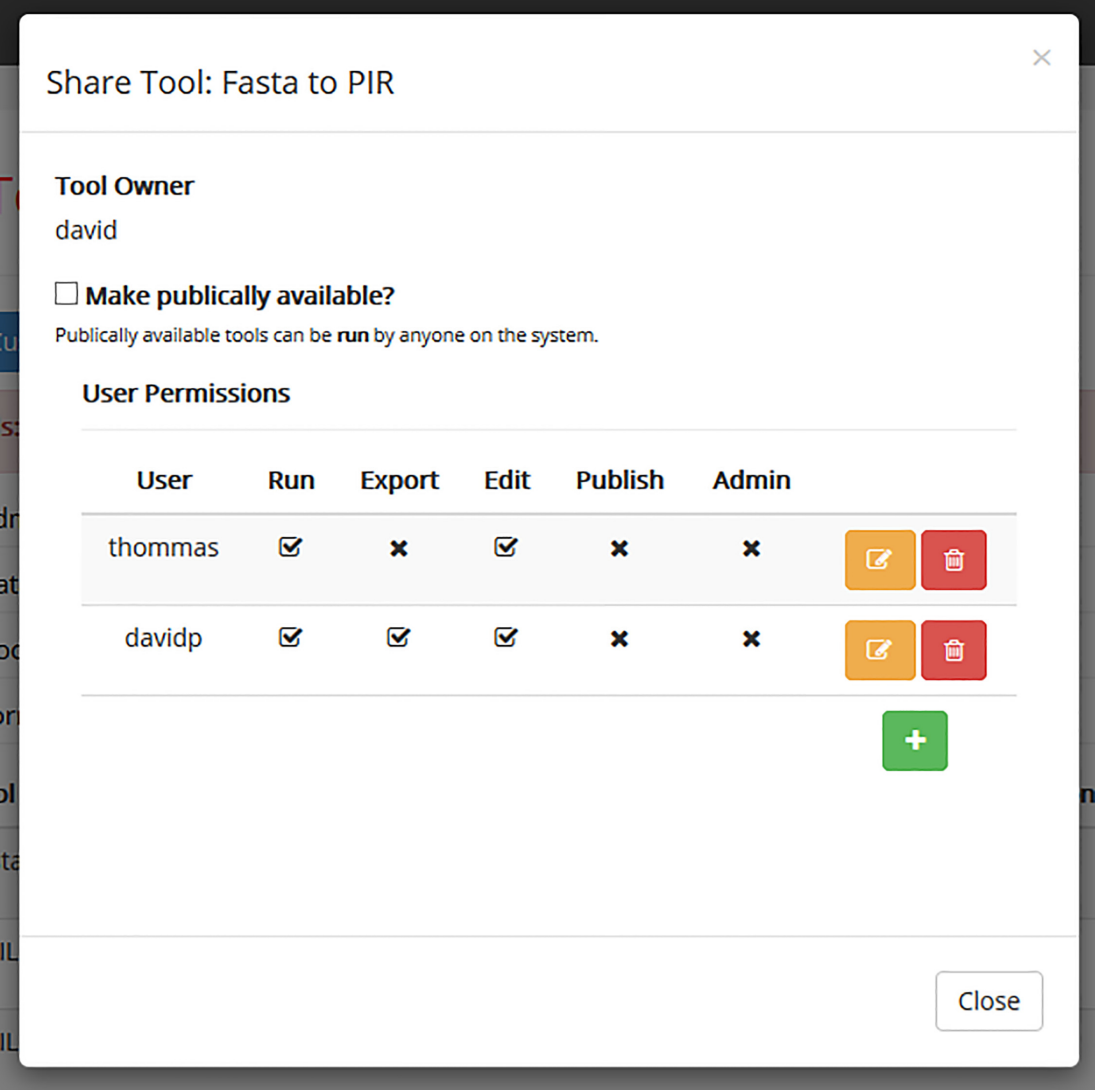
Sharing tools and workflows. The creator of a tool along with any administrators can assign permission to other users on the system.

JMS provides a number of ways to facilitate collaboration between researchers and research groups. Once a user has created a tool or workflow, it can be shared with other users in the system (Fig 9). The creator of the tool or workflow can assign administrator privileges to certain users. Administrators have all the permissions that the creator has except for the ability to remove the creator as an administrator. Administrators can assign permissions to other users. These permissions include the ability to execute the tool or workflow, export and download the tool or workflow to be imported into another instance of JMS, edit the development version of the tool or workflow, and publish a new version of the tool or workflow. Tools and workflows can also be made public, which means that all users on the system will have permission to *run* them.

As alluded to above, JMS provides functionality for workflows to be exported to a compressed file and downloaded. These files can be kept as a backup or distributed to other researchers who can import them into their own JMS instances. In this way, JMS can facilitate collaboration and sharing between researchers working on different systems.

In addition to sharing workflows, JMS allows users to share their jobs and results with other users on the same JMS instance. This means that researchers can work independently on different tasks and later share their results and analyses with one another.

#### 1.7 Cluster Configuration

**Fig 10:**
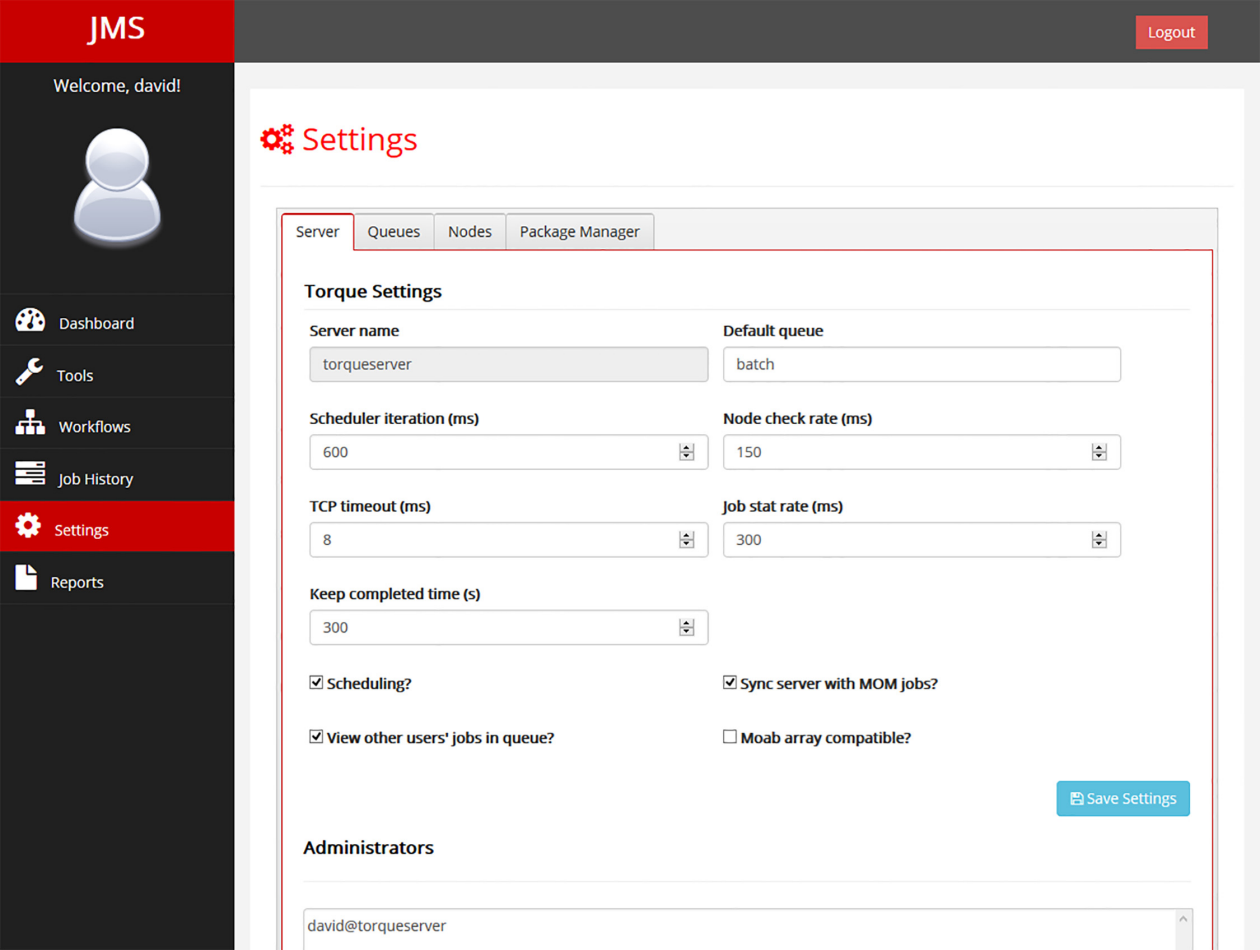
Cluster configuration settings. The settings page can be accessed by both normal users and administrators. Normal users are unable to make any changes on this page, however. Administrators can configure server settings, manage queues and add and remove compute nodes. The exact settings displayed on this page are directly dependant on the underlying resource manager plugin. Optionally, if Ansible support has been configured, administrators can install packages across nodes on the cluster.

In addition to interfacing with the underlying resource manager to provide job management functionality, JMS also provides cluster configuration functionality. This allows administrators to set up and manage queues, configure server settings, and add compute nodes (Fig 10). The exact functionality provided by this page will be dependent on the resource manager plugin being used. In addition, package management functionality can be enabled by enabling Ansible [20] support in the JMS settings.py file. Ansible must be installed on the master node of the cluster.

### Installation

JMS requires a relational DBMS and web server of choice to be installed on the host machine. The system has been tested with SQLite and MySQL DBMSs and the Apache2 web server. A supported resource manager also needs to be installed to facilitate the submission and scheduling of jobs. Lastly, some form of shared storage needs to be accessible across all nodes on the cluster. With the prerequisites installed, JMS can be downloaded from the GitHub repository (https://github.com/RUBi-ZA/JMS). Alternatively, the project can be cloned using a git client (http://git-scm.com/). Once downloaded, JMS can be set up like any Django project. Detailed installation instructions and documentation are available on the JMS GitHub page.

### Security

JMS uses a custom security and authentication system to authenticate directly against the Linux operating system on the master node of the cluster (or any node that jobs can be submitted to the cluster from). As such, users who have an account on the host machine are able to log into JMS using the same username and password. This allows JMS to assume the logged in user’s identity when running processes on the cluster. It also means that these processes then have the same permissions as if the user had submitted the job directly from the command line. Although being able to SSH into the system means that a user will be able to log in to JMS, this is not true the other way around. Accounts can be created that can access JMS, but are not capable of using SSH to log in to the host.

The JMS authentication system is agnostic to the underlying authentication system of the machine it is running on. As such, it supports authentication methods such as LDAP out of the box. In order to achieve this, we have developed the Impersonator server. The Impersonator server is a lightweight web server, which has been developed using the Python Twisted framework [21]. It runs as a separate entity to JMS and is only accessible from the local host i.e. the host that JMS is running on. In addition, valid user credentials must be supplied in every request to the Impersonator server, even if the user is already logged in to the server.

**Fig 11:**
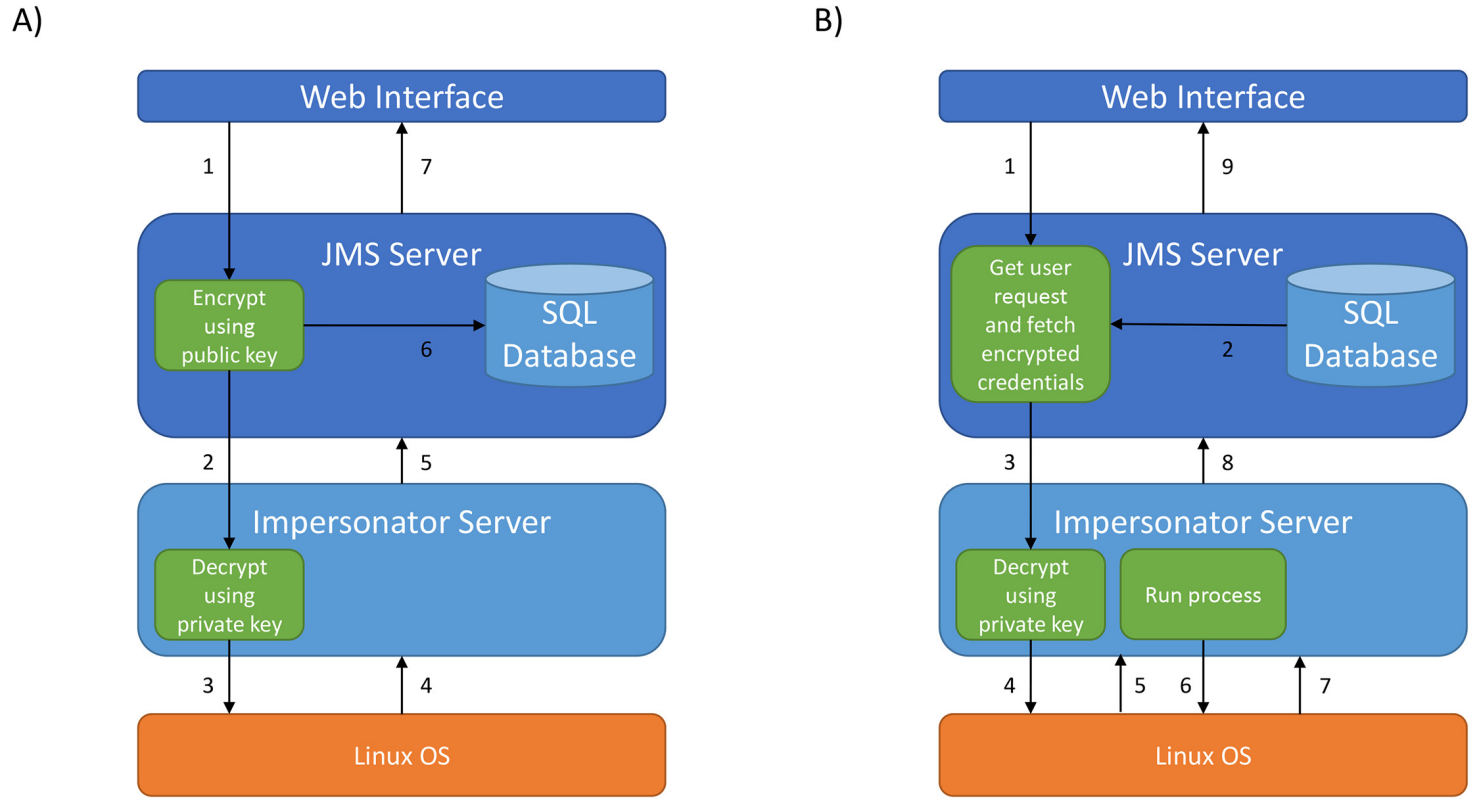
Impersonator Server. A) The login process– 1) credentials are received from the web interface and encrypted using the public key. 2) Encrypted credentials are sent to the Impersonator server where they are decrypted using the private key. 3) Decrypted credentials are used to authenticate the user. 4) The OS responds to the authentication request. 5) The Impersonator server returns the response to the JMS server. 6) If successfully authenticated, the encrypted credentials on the JMS side are stored in the database. 7) The user is redirected to the JMS home page. B) Executing a command– 1) Request is sent from interface. 2) Encrypted credentials are fetched from database. 3) Based on the user request, a command is formulated and sent to the Impersonator server along with the encrypted credentials. 4) The Impersonator server decrypts the credentials and attempts to authenticate the user. 5) The OS responds to the authentication request. 6) A process is spawned in the users name and the command is run. 7) Output from the command is returned. 8) Output from the command is transferred back to the JMS server, which parse is and acts accordingly. 9) A response is sent to the user.

The sole purpose of the Impersonator server is to authenticate users and to receive requests from JMS to launch processes as those users. Communication between JMS and the impersonator server is secured using public key encryption. When a user logs in (Fig 11A), their credentials are immediately encrypted using the Impersonator’s public key. From that moment onwards, user credentials will never be in unencrypted form on the JMS server. The encrypted credentials are encoded and sent to the Impersonator server, where they are once again decoded and then decrypted using the private key, which only the Impersonator server has access to. The Impersonator server can then use these credentials to authenticate the user. On successful authentication, a success message is returned to JMS. The user credentials are then stored in the JMS database in encrypted form. Whenever JMS needs to run a command as a user (Fig 11B), it can send these encrypted credentials, along with the command it needs to run, to the Impersonator server. Once again, the Impersonator will decrypt the credentials to ensure they are still valid and then execute the command. On successful execution, the results are returned to JMS.

## Results & Discussion

A demonstration sever has been made available at https://jms.rubi.ru.ac.za/. Please note that some functionality will not be available on this server.

### 1. Comparison with Other Software

When comparing JMS to other popular tools in the HPC domain, we found five features that distinguished it. The first of these features is the ability to write and edit code directly in the web browser. This feature lets developers create and troubleshoot their workflows directly within their browsers, potentially saving considerable time during the development phase.

The second distinguishing feature was JMS input profiles. When considering whether systems supported input profiles, we required that the users be allowed to create multiple sets of default inputs. Simply allowing a user to set the default inputs for a workflow did not qualify in this regard.

Thirdly, JMS provides administrator users with the ability to manage the cluster from the web interface. This includes configuring server and queue settings as well as adding additional nodes to the configuration. Additional functionality is dependent on the underlying resource manager.

Fourth, a major feature of JMS and one of the original reasons for developing it is its ability to easily and quickly make tools and workflows available via external interfaces. The same code used to generate the interface for a tool within JMS can be used to generate an interface for an external web server.

Lastly, JMS was the only one of the systems tested that provided a comprehensive dashboard. Although most systems provided users with some sort of job queue, JMS is the only system that displays detailed information about the status of the underlying cluster.

A summarised comparison of JMS to other similar and freely available systems is provided in Table 1. Because the aims of JMS do not necessarily align exactly with those of other systems, it is difficult to compare features without seeming slightly biased. For example, in Table 1, JMS is the only system to have cluster configuration features and a dashboard. This hails from the fact that JMS is a combination of a cluster front-end and a workflow management system. A system such as Galaxy is a focused workflow management system and, as such, one would not expect it to have these features. Galaxy does, however, have more extensive workflow features. Notably, it is able to convert job history into a workflow. This provides non-IT experts with an extremely easy means of creating workflows and is something that sets Galaxy apart from other systems. For the most part, shortcomings of JMS amount from the fact that it is a new system. As such, it has only a small user base and library of tools and workflows. As the system matures with time, these facets will be rectified. That said, one of the main goals of JMS is to be a platform for developers to create and house new tools and workflows that will be made public via external web servers. In this regard, JMS stands out from the rest.

**Table 1:** Comparison of JMS with similar tools in the HPC domain.

Features	Galaxy	Ergatis	Taverna	WImpiBLAST	Yabi	JMS
Job management	Yes	Yes	Yes	Yes	Yes	Yes
Workflow management	Yes	Yes	Yes	No	Yes	Yes
File upload/download/view	Yes	Yes	Yes	Yes	Yes	Yes
Development tools	No	No	No	No	No	Yes
Support for multiple resource managers	Yes	No	Not applicable	No	Yes	No
REST API	Yes	No	Yes	No	Yes	Yes
Input profiles	No	No	No	No	No	Yes
Batch jobs	Yes	No	No	No	No	Yes
Cluster configuration	No	No	No	No	No	Yes
Dashboard	No	No	No	No	No	Yes
Make tools public via external web interfaces	No	No	No	No	No	Yes
Job history to workflow	Yes	No	No	No	No	No
Existing user base and external testing	Yes	Yes	Yes	Yes	Yes	No
Library of existing tools and workflows	Yes	Yes	Yes	Yes	Yes	No

As can be seen in Table 1, JMS has both advantages and disadvantages. The lack of a large base of tools and workflows may put off end-users, but the incorporation of development tools may attract developers. There is, however, no reason that JMS cannot be run alongside other systems. In fact, one attractive option may be to run a WMS such as Galaxy alongside JMS on the same cluster. Galaxy is established with many existing tools and workflows and a large user base. However, jobs run on the cluster via Galaxy will still be picked by JMS and included in the JMS job history. The JMS job history stores cluster usage details that are used to generate reports and statistics that may be useful for purchasing and funding purposes. In addition, while users who are more comfortable using Galaxy to run jobs can continue to use Galaxy, developers can use JMS to build tools and workflows and make them public via external web interfaces. In this way, JMS can be seen to complement existing systems.

### 2. Use Cases

Below we provide two structural bioinformatics pipelines as examples of how JMS is currently being used within our group. However, JMS is not limited to running purely scientific workflows. An example currently under development is a submission pipeline for a database of natural compounds. Once it is public, users will be allowed to upload compounds to the database via the JMS-managed workflow.

#### 2.1 Protein-ligand docking

A pipeline for *in silico* docking experiments using Autodock4 [22] has been developed using the JMS workflow tool. The following is a description of how the workflow was designed and how it could be implemented within JMS.

**Fig 12:**
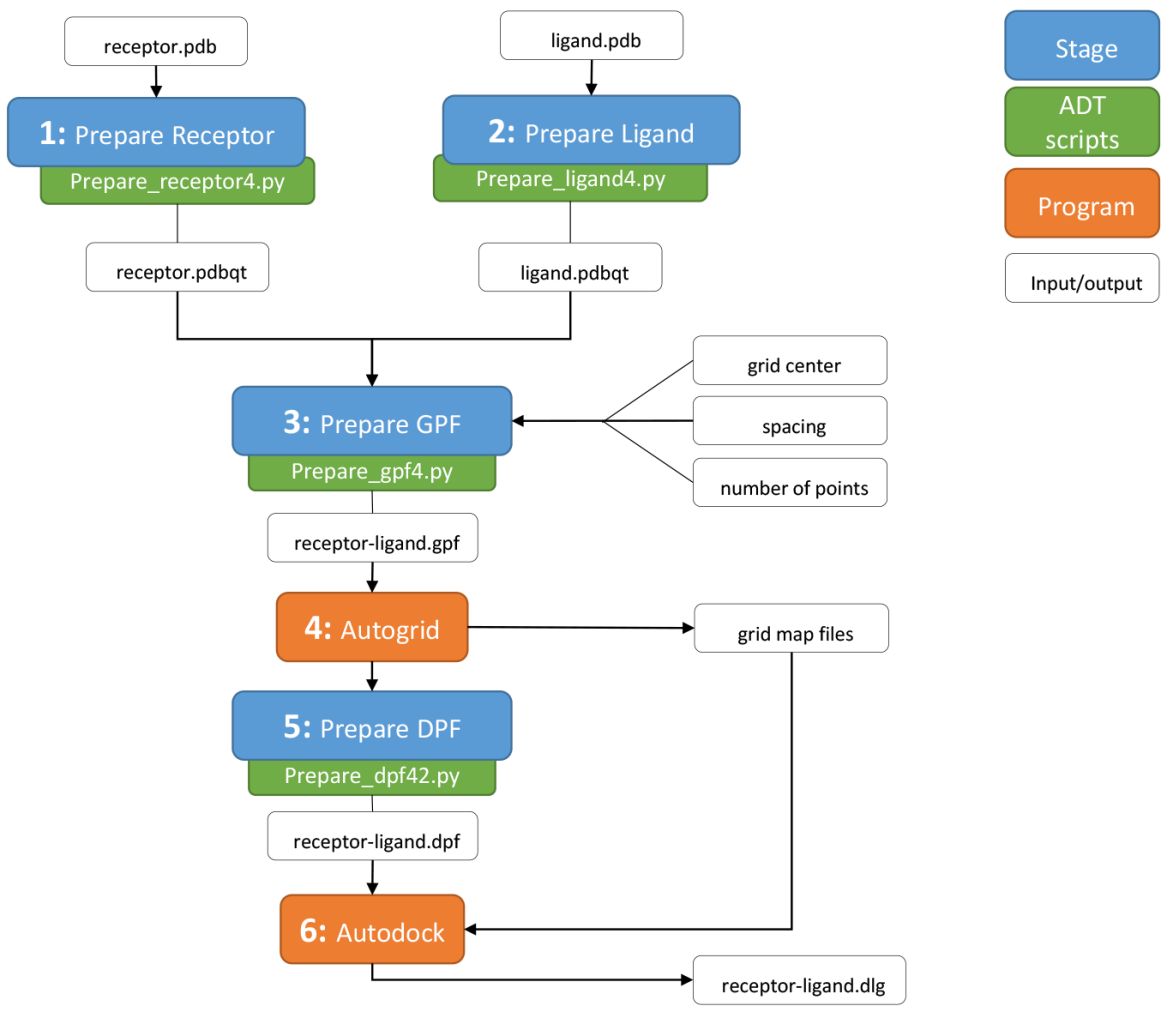
Protein-ligand docking workflow. Schematic flow diagram, showing the logical flow of staged processes in the JMS molecular docking pipeline. Each stage is related to a step required in preparation of small molecule docking with Autodock4. Indicated are each stage as well as the ADT scripts used to execute each respective process. The process requires the protein receptor and ligand of interest in PDB file format, and on completion returns a docking log file containing all docking results.

As previously described, workflow design incorporates custom script uploads. This particular workflow was designed based on Autodock4 scripts made freely available by Autodock Tools (ADT). Virtual docking with Autodock4 is a multi-staged process consisting of 6 specific steps (Fig 12); 1) Receptor preparation, 2) Ligand preparation, 3) Grid map parameterization, 4) Grid map generation, 5) Docking parameterization, and 6) Docking run. All of these steps, bar step 6 are completed in preparation for docking, generating input and reference files. Except for steps 1 and 2, each step must be executed sequentially, each being dependent of the successful completion of the preceding step. Given this logical flow, docking with Autodock4 lends itself towards complete automation, and the JMS workflow design tool and job scheduler provides a suitable platform for which to design such a pipeline.

As indicated in Fig 12, stage 1 and 2, ligand and protein preparation respectively, are independent of one another and as such, both steps are executed simultaneously. On execution the user is prompted to upload PDB files of the protein receptor and ligand respectively. From this initial file upload, the system is instructed to name all output files based on the names of these PDB files. Once receptor and ligand preparation is completed successfully, the execution of step 3 (Prepare GPF) is initiated. This step provides the user with an opportunity to specify their desired docking simulation space, as defined by the gridbox in Autodock4, by prompting the user for three parameters, viz. grid center coordinates, grid spacing (Å), and number of points (npts). These values are written into a grid parameter file (receptor-ligand.gpf) and are required as input for step 4 (Autogrid). Another parameter that can be specified at this stage of the docking process, are the ligand atom types. These have been set to include all atom types, for simplicity’s sake. On successful execution of Autogrid, several grid map files are generated and are required as input for the final docking run in step 6. Prior to the actual docking run, computational docking parameters must be specified. Again the user is prompted to upload their specific parameters. Should the user have no parameter file of their own, a default parameter template stored on the system can be used. Once complete a docking parameter file (receptor-ligand.dpf) is generated and passed as input to step 6. Should every stage prior to docking in the workflow execute successfully, the docking run will be initiated. Molecular docking is a time consuming computational task, and depending on the user specified docking parameters, could take several hours. Progress of the docking can be followed through the *Job History* page, allowing for the monitoring of every stage in the process. Once complete, the user is granted access to a central working directory, where all generated and uploaded files can be viewed, including the all-important docking log file (receptor-ligand.dlg).

This case-study demonstrates how a potential user can execute, accurate and timeous docking experiments, using JMS as a platform from which to run the experiment as a single pipeline. User input is kept to a minimum only requiring the upload of two PDB files and several elementary parameters. By using JMS, the docking process is hugely simplified and, given access to a cluster with a large number cores, docking run times can be greatly reduced, making the JMS Autodock4 molecular docking pipeline not only simple to use, but also very efficient.

#### 2.2 Molecular dynamics

**Fig 13:**
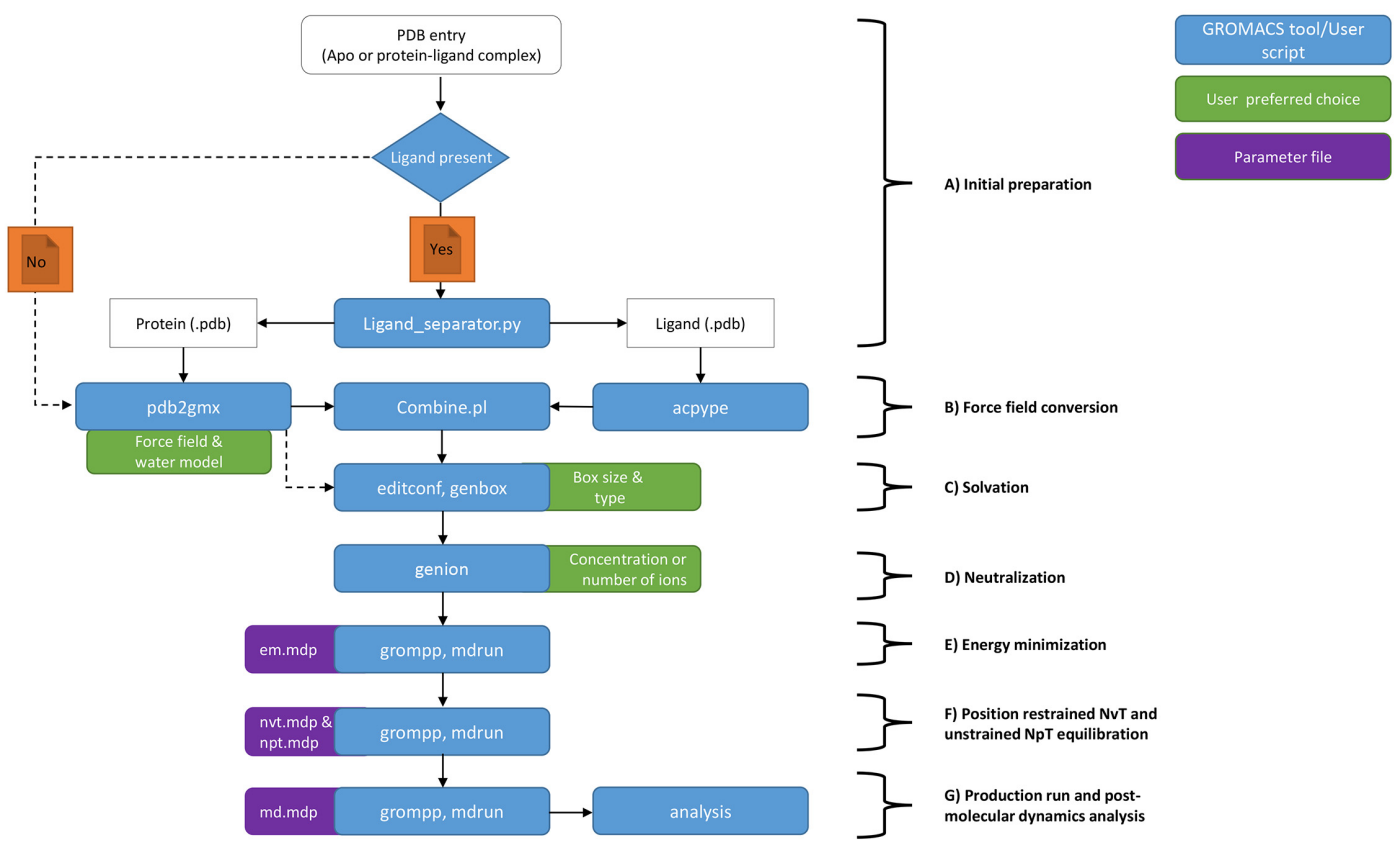
Molecular dynamics workflow. GROMACS is used to perform molecular dynamics simulations on the results from protein-ligand docking.

JMS is used to house a pipeline that utilizes GROMACS 4.5.5 software [23] to perform molecular dynamics (MD) on a protein-ligand complex or apo (protein only) structure (Fig 13). This pipeline can be used in conjunction with the docking pipeline described above or for protein-ligand complexes prepared using other docking programs. The complex is split into separate coordinate files. All line entries in the protein-ligand complex file starting with the word “ATOM” and “HETAM” are transferred into separate protein and ligand pdb files respectively by a python script (Ligand_separator.py) This is necessary as the ligands atoms have to be parameterized using an external tool—ACPYPE [24,25]—for GROMACS to recognize them. Corresponding topology files for both the protein and ligand which represent a static description of all atoms and interactions in a system are generated using the GROMACS utility named *pdb2gmx* (for proteins) and ACPYPE for the ligand. In the case of the protein, the user is prompted to choose a force field (http://md.chem.rug.nl/cgmartini/index.php/blog/265-comparingforcefields) and water model suitable for the system. Using a Perl script, the protein generated coordinate file (.gro) file is combined with that of the ligand. In case of an apo structure, the system bypasses the ACPYPE tool and is taken straight into the *pdb2gmx* tool. The resulting coordinate file is then solvated in a box (type and size specified by user depending on the size of the protein) using the *editconf* and *genbox* tools. From this point, the topology file (.top) is automatically updated to cater for all added molecules in the system. The system is then neutralized by adding counter ions (Na+ or Cl-) via the *genion* tool depending on the net charge. The system energy is subsequently minimized up to a user defined tolerance (emtol) to avoid steric clashes and failure “blowing up”. Parameters such as the type of integrator, maximum stem size (emstep) and number of steps (nsteps) can always be changed depending on user preferences within the em.mdp template using the *grompp* and *mdrun* tools. Other parameters that describe how to find the neighbours of each atom and how to calculate interactions can also be changed depending on the user preference. Using the canonical (NVT) and isothermal-isobaric (NPT) ensembles as per predefined values by the user within the nvt and npt parameter files, the system is equilibrated using the most appropriate algorithms. The equilibrated system is then subjected to a production run whose time length is defined by user within the md.mdp parameter file. The final MD trajectory is analyzed using GROMACS in-house tools that have been automated via Perl and Python scripts. These include RMSD (root mean square deviation), RMSF (root mean square fluctuations), rG (radius of gyration) and protein-ligand interaction fingerprint using LIGPLOT+ (ref). Users may also develop additional tools to analyse the data and incorporate them into the pipeline.

GROMACS usually requires that users input parameters directly into the parameter files and then feed these files to the command-line utilities as input. JMS allows tools to be added that automatically generate these parameter files based on user input. It then generates a web-based input page for these tools. By incorporating these tools at the beginning of the MD pipeline, users can perform molecular dynamics simulations without ever needing to deal with the complex parameter files.

#### 2.3 SANCDB Submission Pipeline

**Fig 14:**
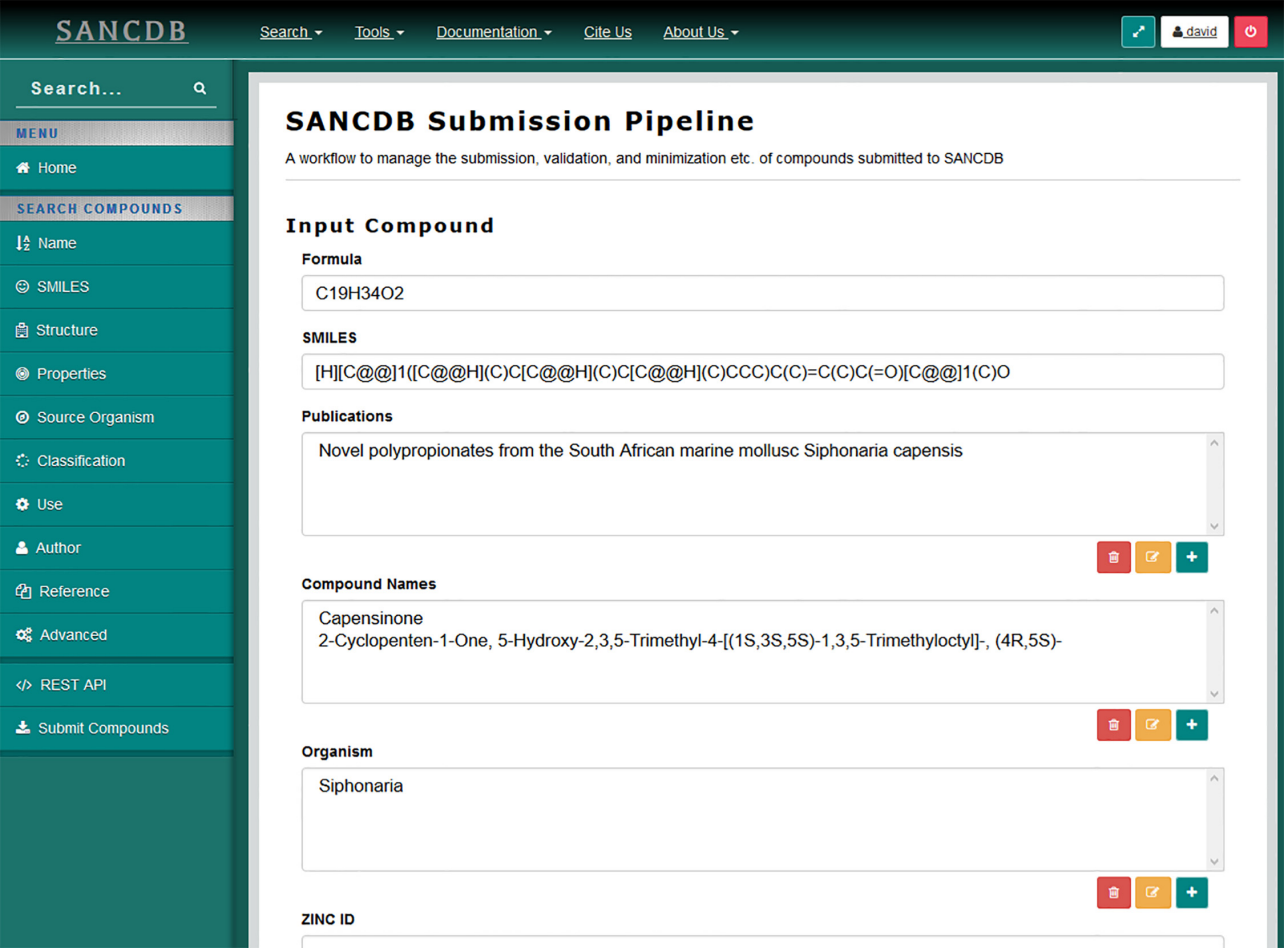
The SANCDB submission pipeline generated interface. The SANCDB interface, including modals and select lists, is generated using the same methods used to generate interfaces within JMS. All submissions are managed via JMS and a detailed job history of the process is stored within the JMS database.

The South African Natural Compounds Database (SANDB) [26] is a publically available database of natural compounds that have been isolated within South Africa. The database is a populated by manually searching through literature to find compounds. This process is labour intensive and, as such, compounds can easily be missed. To ease the burden, a submission pipeline has been developed and incorporated into the SANCDB web interface (https://sancdb.rubi.ru.ac.za). The purpose of the pipeline is to generate a user friendly interface that allows researchers to upload their own natural compounds to the database (Fig 14). Researchers are able to input details such as the compound formula, SMILES [27], publications in which the compound has been isolated, compound names, organisms from which the compound has been isolated, identifiers of the compound in other database such as ZINC [28], DrugBank [29], and ChEMBL [30], classifications of the compound, and the known uses for the compound. The interface to handle all this input is dynamically generated by JMS and is the first stage in the workflow.

The next stage in the submission pipeline is the ‘Format Conversion’ stage. During this stage, the SMILES that were input by the user are used to generate compound files in 3 additional formats, namely, PDB, SDF, and MOL2, using Open Babel [31]. A number of additional calculations and functions are performed during this stage including calculating properties such as the atomic mass of the compound and generating a 2D image of the compound to be displayed on the website.

The final stage of the pipeline is the ‘Minimization’ stage. In this stage, the PDB file generated during the previous stage is minimized using GAMESS [32]. Both the original and minimized version of the compound are made available in the SANCDB website.

Once the submitted compound has been through all the required stages, it is primed and ready to be manually checked by a database curator. If everything worked, the compound can be made publically available. All the steps in this process are run via JMS and as such, a complete job history is kept.

## Future Work & Conclusion

JMS provides three distinct categories of functionality. Firstly, it provides a web interface to an HPC resource manager with the aim of making the resource manager much easier to work with. In order to achieve this, it employs the use of resource manager plugins which interface with the underlying resource manager to provide job and cluster monitoring and management functionality.

Secondly, JMS provides a fully functional workflow management system, which integrates directly with the resource manager. The WMS provides advanced scheduling and dependency abilities, input profiles, batch jobs, and, we believe, a far easier way of building and managing tools than existing systems provide. Workflows can then be created via an intuitive drag and drop interface, similar to what is used in Galaxy.

Lastly, JMS allows workflows to be integrated into other, external websites via its RESTful web API. This functionality is demonstrated via the use of JMS to provide a submission pipeline for SANCDB. Using JMS, researchers are able to create workflows via the click-based web interface and then easily integrate these workflows into their own sites. This functionality substantially reduces the effort of creating web interfaces for computational pipelines. Further, we believe that JMS will facilitate the sharing of information and computational techniques within H3Africa collaborations and help with the setting up and managing of cluster related infrastructure. The inclusion of resource manager administration features means that JMS is useful to both casual users, who simply want to run jobs over their cluster, and system administrators, who can use it for monitoring and managing the cluster.

JMS is still under active development, and there are a number of features that will soon be introduced. Currently, workflows can only be made up of command-line utilities and custom scripts. A feature will soon be added that allows a workflow to be made up of other workflows in addition to script and command-line utilities. This will reduce duplication of effort when creating large workflows that require results that can be obtained from existing workflows.

Currently, when workflows are exported, a compressed file is created containing the required custom scripts and details needed to recreate the workflow on another system (excluding tools that need to be installed on the system). This file can then be manually imported into another JMS instance via the workflow page. In future, users will be able to transfer workflows directly from one instance of JMS to another. The only input that will be required is the URI-based address of the system to transfer to as well as the user on that system who will gain ownership of the workflow. That user will then receive a notification that a workflow has been sent to them and will simply need to accept the transfer. We hope that this feature will help facilitate collaboration between groups. In line with this, we will also develop a repository of tools and workflows, similar to what is found in Galaxy. Users will be able to upload their tools and workflows to the repository, where they can be made available for download by any connected JMS instances.

Long-term plans for JMS will, for the most part, be focused on adding and improving administrative features. Currently, JMS must be installed on the master node of a cluster. In future, JMS will be extended to allow it to manage multiple clusters in remote locations. In line with this, we will also provide advanced scheduling functionality that will let users schedule workflows across multiple clusters. This will allow JMS to operate over and manage whole grids of clusters.

In addition to this, we will develop JMS apps for smartphones that will allow normal users to monitor and submit jobs from their mobile devices as well as allow admin users to receive status notifications and requests for resources. Although the JMS interface has been design to be responsive–it adjusts its layout according to the size of the screen it is being viewed on—mobile apps have the added benefit of allowing notifications to be sent to the user even if that user is not currently using the app. This will allow administrators to instantly respond to notifications and/or requests for additional resources. The mobile apps will also keep administrators up-to-date on the current state of the cluster and notify them when, for example, a node goes offline.

Lastly, because we store snapshots of jobs at each stage, a single job can end up taking a lot of storage space. In future, JMS will allow admin users to set up an automatic backup and archiving solution, whereby old jobs are compressed and copied to an external location.

In conclusion, JMS aims to provide a user-friendly interface that allows both novice and advanced users to benefit from the advantages of high performance computing. The system has been open-sourced and is available on GitHub at https://github.com/RUBi-ZA/JMS. JMS has been designed to be extendible and, as such, we encourage any and all community contributions to the project.
